# Animal obesity: causes, consequences and comparative aspects

**DOI:** 10.1186/s13028-016-0237-0

**Published:** 2016-10-20

**Authors:** Bodil Ström Holst, Malin Hagberg Gustavsson

**Affiliations:** Department of Clinical Sciences, Swedish University of Agricultural Sciences, Box 7054, SE-750 07 Uppsala, Sweden

The prevalence of obesity has increased in the last decades, both in humans and in several animal populations [1]. The increasing obesity in people is one of the most severe global health concerns of our time, leading both to increased disease prevalence and to reduced fertility, and the situation is similar in several animal species.

Changes in prevalence of obesity in sports- and companion animals mirror the increases in the human population. Similar environmental factors, such as inadequate exercise and excess intake of highly digestible food, are implicated as causes, besides genetic components. Various problems are associated with obesity. Diabetes is one of the most common endocrine disorders in cats, as is the metabolic syndrome in horses. There is also a clear link between body condition and fertility. This is a potential problem not only in companion animals, but also in production animals, and for example over conditioned cows show altered metabolism.

To effectively prevent the occurrence of obesity and increased morbidity in animals, a holistic view should be applied. This requires multidisciplinary collaboration combining the expertise and skills from different fields. Comparative studies are also needed. Comparative studies on prevention and pathogenesis of obesity, as well as diagnosis and therapy of related diseases, enable a better understanding of underlying basic mechanisms. To promote and facilitate multidisciplinary and comparative research, events where scientists can meet and share ideas are needed.

At the multidisciplinary congress Animal Obesity — causes, consequences and comparative aspects, held at the Swedish University of Agricultural Sciences, 14–16 June 2016, researchers with a common interest in different issues related to animal obesity established new national and international contacts. The congress brought together researchers within veterinary medicine, veterinary nursing, microbiology, animal science, ethology, biology and genetics. Several topics were included, such as aetiology, diagnosis and treatment of diseases related to obesity, including diabetes mellitus in cats and the metabolic syndrome in horses, reproductive disorders, and the link between obesity and inflammation.

The multidisciplinary approach was reinforced by the congress not having parallel sessions. There were plenary lectures of invited speakers, short oral research presentations, poster displays and a summarizing panel discussion on how to deal with future challenges. Time for discussion and social interaction was generous.

Presenting delegates were offered the opportunity to submit manuscripts to the present peer reviewed *Acta Veterinaria Scandinavica* supplement; Animal Obesity — causes, consequences and comparative aspects: current research. All publication fees were sponsored by the congress organizer, the research platform Future Animal Health and Welfare at the Swedish University of Agricultural Sciences.
